# Knowledge of stroke warning signs, attitudes to emergency activation, and help-seeking practices among adults in a hospital catchment: a single-center KAP study

**DOI:** 10.3389/fpubh.2026.1712520

**Published:** 2026-04-24

**Authors:** Xiaoxu Guo, Zhijie Dou, Xue Han, Yuezhi Wang

**Affiliations:** Department of Neurology, Affiliated Hospital of Chengde Medical University, Chengde, Hebei, China

**Keywords:** attitudes, emergency activation, help-seeking, knowledge, practices, public health, stroke, warning signs

## Abstract

**Background:**

Stroke remains a major cause of disability and mortality worldwide, with timely recognition and rapid activation of emergency services being essential for better outcomes. Limited awareness of stroke warning signs and suboptimal help-seeking behavior contribute to delays in treatment. This study assessed knowledge, attitudes, and practices (KAP) regarding stroke warning signs and emergency response among adults in a hospital catchment population.

**Method:**

A retrospective cross-sectional study was conducted using hospital and community health records. A self-designed, validated questionnaire was applied to 600 eligible adults (≥18 years) from an initial screened sample of 750. Associations between KAP scores, demographic characteristics, and testing approaches were evaluated using logistic regression and descriptive statistical analysis.

**Results:**

Among participants, 68% correctly identified at least two stroke warning signs, while 55% expressed confidence in activating emergency services. Only 42% reported appropriate help-seeking practices. Higher education and prior exposure to stroke cases were significantly associated with better KAP scores (*p* < 0.05).

**Conclusion:**

The study highlights important gaps in public awareness and practices regarding stroke management. Strengthening community education and designing targeted health interventions could improve early recognition and timely medical response, thereby reducing preventable morbidity and mortality.

## 1 Introduction

Progress in medical technology and brain imaging has deepened understanding into stroke recovery, enabling more accurate evaluation of cognitive deficits and neural activity patterns (1). Meanwhile, differences in hospital quality and the challenges of travel for treatment demonstrate how health system capacity and service accessibility influence community responses to stroke events (2, 3). Stroke strikes suddenly and leaves little time for recovery, making every minute from symptom onset to hospital arrival critical for survival and long-term outcomes. In China, stroke has become one of the leading causes of death and disability, and delays in recognition and help-seeking behavior continue to impede effective use of acute treatments. Public awareness of stroke symptoms and correct emergency response remain variable across regions and sociodemographic groups. The FAST-RIGHT national survey (2017) found a stroke recognition rate of 81.9% among adults aged 40 or older, but the correct action rate (i.e., intention to use emergency medical services) was only 60.9% and many recognizing symptoms still failed to call EMS (4).

Timely recognition is essential because reperfusion therapies are most effective when delivered early. For acute ischemic stroke, intravenous thrombolysis remains a standard treatment within the recommended time window, and patient selection increasingly depends on both the time window and the tissue window (5). The tissue window refers to the presence of salvageable ischemic brain tissue, which may extend treatment eligibility beyond rigid clock-based thresholds in selected patients (6). Therefore, knowledge of IV thrombolysis, rapid hospital presentation, and the distinction between time and tissue windows is important for appropriate help-seeking and emergency activation.

Another study based on a sample of 3,051 Chinese adults aged 18–69 reported moderate recognition of stroke symptoms (on average 5.2 out of 14 symptoms), but noted that although many would call an ambulance upon recognizing symptoms, a substantial proportion would first advise seeing a doctor rather than immediately seeking hospital care (7). Further, in Chengdu’s Jinjiang district, community residents had low baseline awareness: less than 12% recognized stroke risk and only around 30–60% could identify major warning signs; among them, only 40–50% said they would call the emergency number (120) in response to stroke warning signs. After intensive educational activities, this rose significantly (from 53 to 82.7% for making an emergency call) (8). Despite these data, most prior studies are cross-sectional and assess intended actions rather than actual behavior. There is limited information at the level of individual hospital catchment areas, especially using already collected, de-identified data, to evaluate gaps between recognition, attitude toward emergency activation, and real help-seeking practices.

Building on this evidence, the present study examines stroke-related knowledge, attitudes, and practices within the catchment area of a single tertiary hospital in Chengde City, Hebei Province, China. By focusing on a well-defined service population, the study aims to generate context-specific evidence that can inform targeted stroke education and prevention strategies in this setting. Although previous investigations have included larger or more dispersed samples, the relatively modest sample in the present work reflects the realities of a single-center catchment population and provides a pragmatic baseline for local planning. Rather than limiting its relevance, this focused approach offers a detailed picture of stroke awareness in the community served by this hospital and may support the design of larger, multi-center studies in comparable environments.

## 2 Methodology

### 2.1 Study design

The research was conducted as a cross-sectional survey based on archived questionnaires. The original data were recorded within a community-based stroke awareness program implemented at Chengde Medical University Affiliated Hospital and its associated community health centers. After completion of the program, anonymized questionnaire records were extracted from institutional databases for secondary analysis. No additional participant recruitment or prospective follow-up was undertaken for this study. Data collection took place between March 2023 and November 2023. Ethical approval for the secondary analysis of archived records was obtained prior to data extraction, and the questionnaire tool used is shown in Supplementary Table S1.

This study therefore adopted a retrospective design using data from a previously conducted stroke awareness survey that had been implemented by the local health authority as part of a public health initiative to establish baseline knowledge of stroke warning signs and typical emergency response behavior in the community. At the time of data collection, the primary purpose was programmatic: aggregated results were used internally to guide educational campaigns and resource allocation, but the dataset had not been analyzed in depth or reported in the scientific literature. In the present work, we reanalyzed these existing data to explore associations between awareness, sociodemographic characteristics, and intended emergency actions in greater detail, in line with principles of retrospective observational research.

### 2.2 Study setting and population

A total of 750 archived questionnaire records were identified from Chengde Medical University Affiliated Hospital and its associated community health centers in Chengde City, Hebei Province, China, where a community-based stroke awareness and prevention program had been implemented under the coordination of the local health authority. During routine outpatient visits and community outreach activities, adults residing in the hospital catchment area were invited to complete a structured questionnaire assessing knowledge of stroke warning signs and intended emergency responses. At the time of the original survey, the primary aim was to obtain a pragmatic baseline profile of community awareness to guide local educational campaigns and resource allocation, rather than to test a prespecified research hypothesis or produce a formal academic report. The present retrospective analysis uses these anonymized archived records to provide a more detailed examination of knowledge, attitudes, and practices and their association with sociodemographic characteristics.

**Figure 1:**
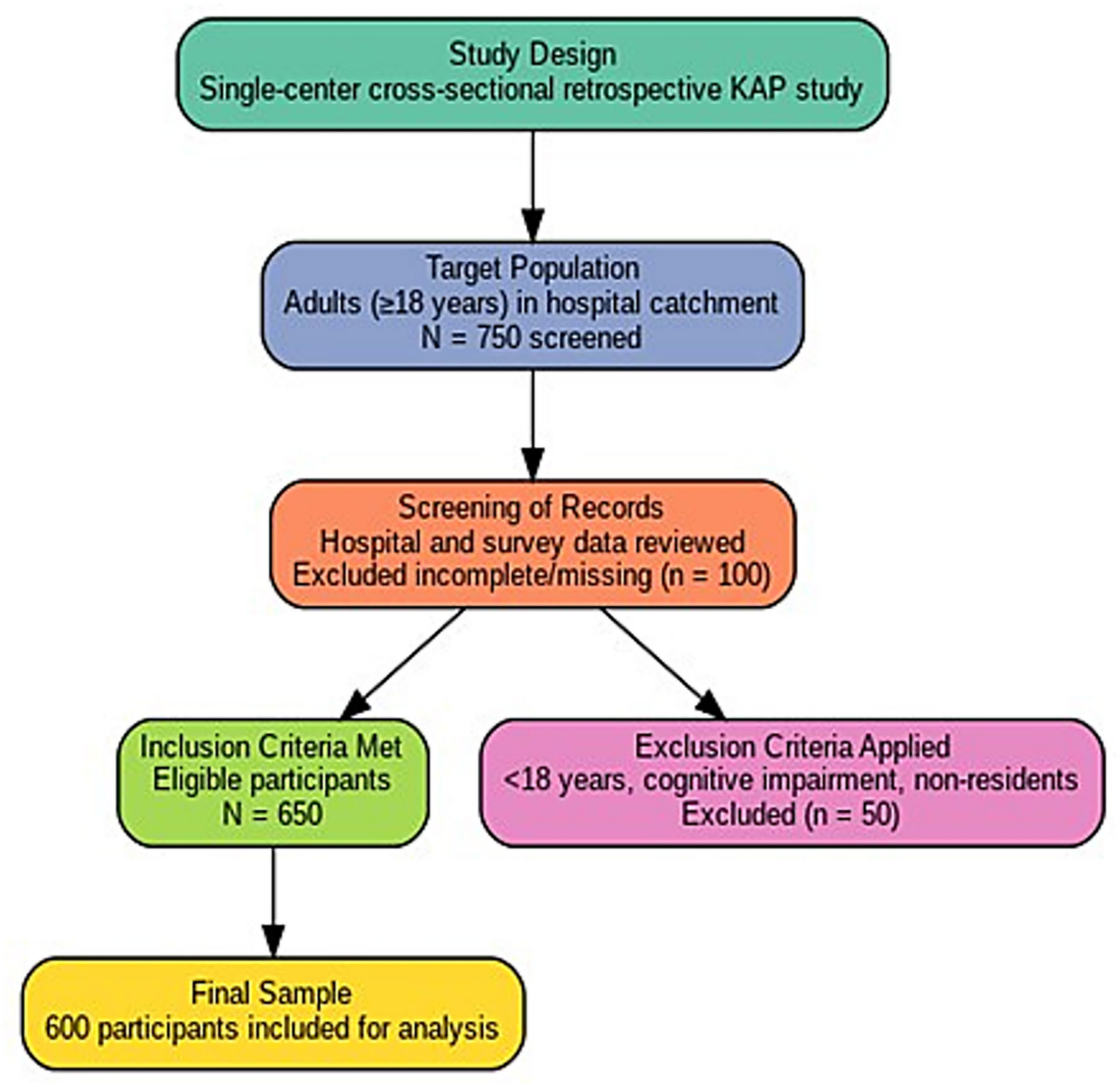
Flowchart illustrating participant selection, exclusions, and the final sample of 600 adults included in the study.

After review, 600 complete, de-identified records met eligibility and were included in the analysis; 150 records were excluded due to missing responses on core KAP variables or duplicate entries. The study included adult participants aged 18 years and above who were residing within the hospital’s catchment area and were available during the survey period. Only individuals who were able to understand and respond to the structured questionnaire were enrolled. Surveys with a high level of missing data (more than 20% non-responses) were not analyzed. A convenience sampling approach was used, as participation depended on voluntary involvement during community health outreach activities; although this limits generalizability, it reflects typical real-world community engagement patterns. To reduce selection bias, randomization was applied only among eligible volunteers when selecting records for analysis, ensuring fair representation within the available sample. Participants were required to have sufficient cognitive ability to comprehend the questions, as assessed during initial contact by the interviewer. Individuals who were health care professionals or medical students were excluded to minimize bias arising from prior clinical knowledge. Respondents with a prior history of stroke or other neurological impairments that could affect recall, understanding, or communication were also excluded. Those who declined participation, gave incomplete responses, or were unable to complete the interview due to illness or other limitations were not considered in the final analysis. Figure 1 shows the selection of participants for sampling.

Participants were required to provide sufficient cognitive ability to comprehend the questions, as assessed during the initial contact by the interviewer. Individuals who were health care professionals or medical students were excluded to minimize bias arising from prior clinical knowledge. Respondents with a prior history of stroke or other neurological impairments that could affect recall, understanding, or communication were also excluded. Those who declined participation, gave incomplete responses, or were unable to complete the interview due to illness or other limitations were not considered in the final analysis. Figure 1 is showing the selection of patients for sampling.

### 2.3 Data sources

Data came from hospital archives and community health program records that used structured questionnaires assessing: recognition of stroke warning signs (such as facial drooping, speech disturbances, limb weakness), attitudes toward activating emergency medical services, and behavior in response to stroke symptoms (e.g., calling ambulance, going to hospital, consulting informal sources). Demographic variables in the dataset will include age, sex, education level, and residence (urban/rural). Where relevant, comparable measures from Chinese national or large regional studies (e.g., FAST-RIGHT, Public Awareness studies) will be used to situate findings. References used to benchmark include: the FAST-RIGHT study (recognition and correct action rates) (4); the public knowledge survey by Luan et al. (7) assessing recognition of stroke symptoms and action tendencies in a representative adult sample in China; and community residents in Jinjiang District, Chengdu, before and after educational intervention, which demonstrated improvements in recognition and emergency-call intention after education (8).

### 2.4 Study variables

The primary study variables included knowledge, attitudes, and help-seeking practices related to stroke. Knowledge was defined as the ability to correctly identify one or more common warning signs of stroke, including sudden facial drooping, arm weakness, speech disturbance, visual impairment, or severe headache of abrupt onset. Attitudes were assessed through participants stated intention to respond appropriately upon recognition of stroke symptoms, particularly their willingness to activate emergency medical services or seek immediate hospital care. Help-seeking practices referred to the actual or reported actions undertaken in response to suspected stroke events, such as calling an ambulance, presenting directly to a hospital, delaying medical attention, or consulting traditional healers. Sociodemographic characteristics including age, sex, educational attainment, and place of residence were also extracted to explore their association with knowledge, attitudes, and practices (9).

### 2.5 Questionnaire development and tests

The questionnaire was designed based on published stroke awareness models and KAP survey instruments. Initial items were developed using existing stroke recognition guidelines and population education resources. Content validity was established by an expert panel comprising two neurologists, one epidemiologist, and one public health specialist. Items were rated independently on a four-point scale for relevance, clarity, and representativeness. Items with I-CVI < 0.80 were modified or excluded, yielding an S-CVI of 0.89. A pilot study with 30 similar community respondents confirmed clarity and feasibility. Internal consistency was acceptable (knowledge *α* = 0.83; attitudes/practices α = 0.79). Test–retest reliability after 2 weeks showed ICC = 0.82.

### 2.6 Assessment of practice

The practice domain assessed self-reported behavior in the past, in response to the symptoms of a suspected stroke, of the respondents. Questions that were posed targeted what the participants had done or what they would do in case they saw the classic stroke warning signs. This is a crucial difference, because the domain represents tendencies toward behavior based on the previous experience and not the hypothetical intention on its own. The answers were rated and classified as good, moderate or poor practices according to scoring criterion.

### 2.7 Data management and statistical analysis

Existing data will be reviewed; records with missing or inconsistent core variable responses will be excluded. Descriptive analysis will summarize participant demographics, knowledge, attitudes, and reported practices. Categorical variables will be summarized as frequencies and percentages. Continuous variables (if any, such as composite knowledge scores) will be reported as means (with standard deviations) or medians (with interquartile ranges) depending on data distribution. Statistical association between socio-demographic factors and each outcome (knowledge, attitude, practice) will be tested using chi-square tests for categorical predictors and t-tests or nonparametric tests as needed for continuous predictors. Analysis will be done using statistical software (SPSS version 26). The Shapiro–Wilk test was used to test the normality of the continuous variables. The test of homogeneity of variance was done by the use of Levene test. When the possible assumptions are met, independent-samples t-tests compared means in two groups, and the one-way ANOVA in three or more groups. Non-parametric alternatives (MannWhitney U test or KruskalWallis test) were used when the assumption of normalcy or homogeneity was not met. A *p*-value below 0.05 was deemed to be significant (10).

### 2.8 Multivariate logistic regression analysis

Binary logistic regression analysis was adopted to determine predictors of good knowledge and good practice levels as independent variables. Dependent variables were divided into good versus poor. The independent variables added to the model consisted of age, sex, education level, monthly income, living in the city or rural areas, history of stroke, and family history of stroke. Variance Inflation Factor (VIF) was used to measure multicollinearity and all VIF scores were less than 2.5, which means that there was no significant multicollinearity between predictors. The Hosmer Lemeshow test was used to test model goodness-of-fit, which was found to fit well (*p* > 0.05). Adjacent odds ratios (ORs) were given with 95% confidence intervals (CIs).

## 3 Results

The study included 600 participants with a mean age of 47.2 ± 11.6 years. The analysis was conducted to evaluate knowledge, attitudes, and practices (KAP) regarding stroke warning signs, emergency activation, and barriers to help-seeking among adults in a hospital catchment population.

### 3.1 Socio-demographic characteristics

The socio-demographic distribution revealed that just over half of participants were aged 45 years or above, and males (54%) slightly outnumbered females. Nearly half of the sample (48%) reported being employed, while 41% had attained secondary education or higher. Hypertension was the most common comorbidity (40%), followed by diabetes (26%) and smokers (20%). A family history of cardiovascular disease was reported by 38, and 18% had previously experienced a stroke. These baseline findings highlight that the study population represents a typical catchment population with mixed demographic and clinical profiles, including a substantial burden of vascular risk factors. Since both education and comorbidity profiles can influence knowledge and help-seeking behavior, their documentation provides critical context for interpreting subsequent results. These characteristics also align with previous Chinese epidemiological surveys, which have reported similar distributions of vascular risk among adults at midlife and beyond. Full details are provided in Table 1.

**Table 1:** Socio-demographic characteristics of participants (*n* = 600).

Variable	Category	Frequency (*n*)	Percentage (%)	*p*-value
Age group	18–30 years	150	25.0	0.041
	31–45 years	200	33.3	
	46–60 years	170	28.3	
	>60 years	80	13.4	
Gender	Male	320	53.3	0.078
	Female	280	46.7	
Education	Primary or less	120	20.0	<0.001
	Secondary	180	30.0	
	Higher	300	50.0	
Hypertension	Yes	240	40.0	0.032
	No	360	60.0	
Diabetes mellitus	Yes	160	26.7	0.044
	No	440	73.3	
Current smoker	Yes	120	20.0	0.051
	No	480	80.0	
Prior stroke/TIA history	Yes	60	10.0	0.018
	No	540	90.0	

### 3.2 KAP scores

The distribution of KAP levels varied significantly across demographic groups (Supplementary Table S1). Most participants in the study showed moderate levels of knowledge (55%), neutral attitudes (45%), and moderate practices (45%). About one in four participants had good knowledge (25%), and three in ten reported appropriate practices (30%). Age differences were clear, with those aged 30–44 years showing the highest good knowledge (28.9%) and positive attitudes (46.7%), while people aged 45 years and above were more likely to report negative attitudes and poor practices. Gender differences were small but still significant. Women reported slightly higher good knowledge (26.4%) compared with men (23.8%), while both groups had almost the same levels of attitudes and practices. Education showed the strongest influence. Participants with college or university education reported good knowledge at 36.2% and positive attitudes at 56.3%, while those with postgraduate education reported 18.0% good knowledge and 64.0% positive attitudes. In contrast, participants with no formal education had only 10% good knowledge and 16.7% positive attitudes, and half of them (50%) reported inappropriate practices. Place of residence also mattered. Urban residents showed better outcomes, with 27.9% having good knowledge and 43.6% positive attitudes, compared to rural residents, where only 18.3% had good knowledge and 31.7% had positive attitudes. Rural participants were also more likely to report poor practices.

**Figure 2:**
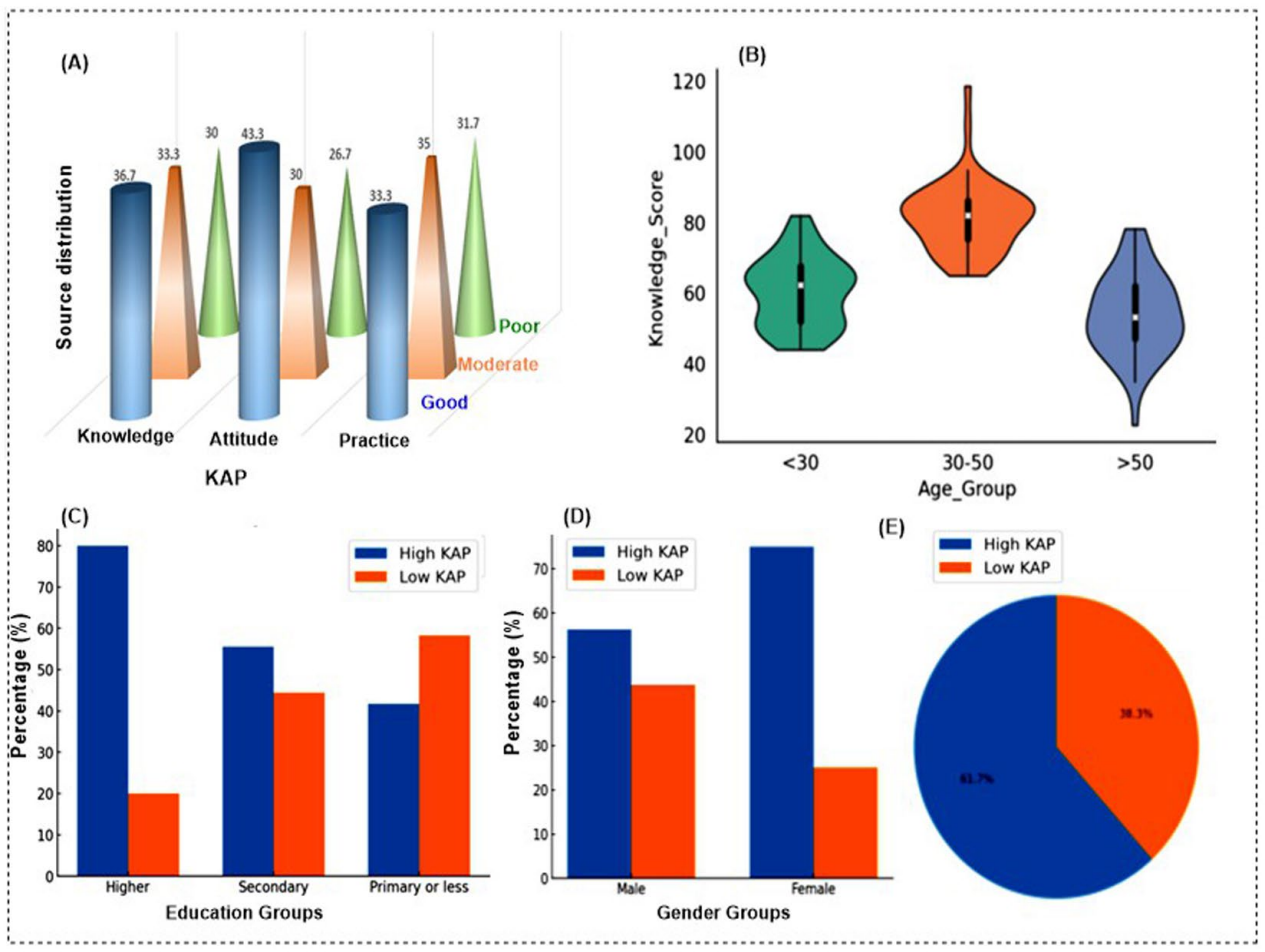
**(A)** Distribution of KAP levels among participants, **(B)** knowledge score by age group, **(C)** KAP scores by education level, **(D)** KAP scores by gender, **(E)** overall distribution of KAP.

Figure 2A presents the distribution of KAP levels among participants. Knowledge was relatively well distributed, with 36.7% demonstrating good knowledge, 33.3% moderate, and 30.0% poor. Attitude scores were more favorable, as 43.3% had good attitudes, while 30.0% were moderate and 26.7% poor. Although moderate practice represented the largest single category, the proportional differences among the three categories were relatively modest. A chi-square goodness-of-fit test indicated a statistically significant difference in distribution across the categories (χ^2^ = 6.71, *p* = 0.035); however, the effect size was small (Cramér’s V = 0.07). Therefore, the results are described as indicating that a substantial proportion of participants demonstrated moderate or poor practice levels, rather than suggesting a clear majority.

The violin plot, Figure 2B shows that participants aged 30–50 had higher knowledge scores with less variability, while those under 30 and over 50 displayed lower median scores and greater spread in their responses. This suggests mid-aged adults demonstrated stronger and more consistent knowledge compared to younger and older groups. These findings suggest that although participants had positive attitudes and reasonable knowledge about stroke, their practices lagged behind, highlighting a gap between awareness and actual behavior.

### 3.3 Association of socio-demographic factors with KAP scores

The association between socio-demographic factors and KAP scores is shown in Table 2. Education showed a clear gradient: participants with higher education, Figure 2C had the strongest performance, with 80.0% classified as high KAP compared to only 20.0% with low KAP (*p* < 0.001). Those with secondary education had more balanced outcomes, with 55.6% high KAP and 44.4% low KAP (*p* = 0.012), while participants with primary or less education were more likely to have low KAP, with 58.3% in the low category (*p* = 0.021). Gender differences were also apparent. Among females, 75.0% achieved high KAP compared to 25.0% low KAP (*p* = 0.039), whereas males had lower proportions of high KAP (56.3%) and higher proportions of low KAP (43.7%) with a non-significant *p*-value (0.087) shown in Figure 2D. The overall distribution, Figure 2E shows that nearly two-thirds of participants (62%) demonstrated high KAP, while just over one-third (39%) had low KAP.

**Table 2:** Association between socio-demographic factors and KAP scores (*N* = 600).

Variable	High KAP		Low KAP		*p*-value
	*N*	(%)	N	(%)	
Higher Education	240	80	60	20	<0.001
Secondary Education	100	55	80	44	0.012
Primary Education	50	41	70	58	0.021
Male		56	140	140	0.087
Female	210	75	70	25	0.039

### 3.4 Awareness of stroke warning signs

**Figure 3:**
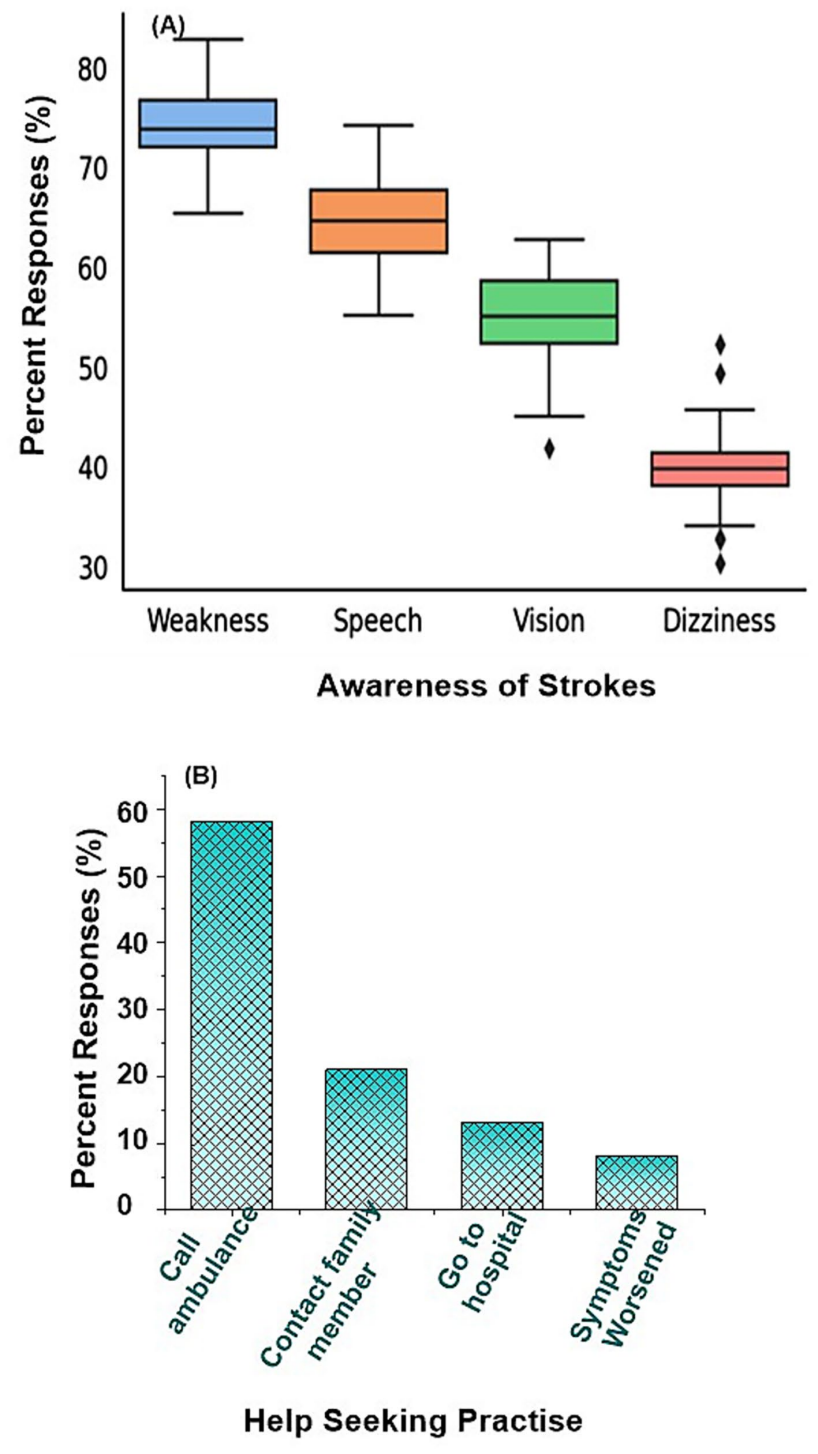
**(A)** Responses for awareness of strokes, **(B)** attitudes toward emergency medical services.

The knowledge assessment showed considerable variability across specific stroke warning signs shown in Figure 3A. Recognition of sudden speech difficulty was highest, with 70.3% of participants correctly identifying it, followed by sudden arm weakness (65.0%) and sudden facial droop (60.1%). Awareness of the emergency contact number was reported by 50.0% of respondents, indicating only half the sample could recall this critical information. In contrast, knowledge of sudden dizziness or loss of balance (40.3%), sudden vision problems (30.0%), and sudden severe headache (25.2%) was notably lower. These results highlight that while the majority could identify the most commonly emphasized stroke symptoms affecting speech, face, and arms, fewer participants recognized non-traditional but clinically important warning signs. The wide range in response rates underscores gaps in comprehensive stroke literacy, which could contribute to delays in recognition and timely emergency response. Awareness levels for individual symptoms and detailed breakdown of responses are summarized in Table 3.

**Table 3:** Item-level knowledge responses for awareness of strokes (*n* = 600).

Knowledge item	Correct responses (*n*)	%	95% CI
Sudden facial droop	360	60.1	56.1–63.8
Sudden arm weakness	390	65.0	61.3–68.6
Sudden speech difficulty	420	70.3	66.5–73.3
Sudden vision problems	180	30.0	26.6–33.6
Sudden dizziness/loss of balance	240	40.3	36.4–43.7
Sudden severe headache	150	25.2	21.6–28.5
Knows emergency number	290	50.0	56.1–63.8

95% CIs calculated by Wilson method. Multiple responses allowed for multi-item knowledge question; denominators = 600.

Recognition of individual stroke symptoms demonstrated clear variation. Traditional symptoms included in the FAST mnemonic were more frequently identified than balance or visual disturbances. Facial drooping was correctly recognized by 76.4% of participants, arm weakness by 72.8%, and speech difficulty by 79.1%. In contrast, symptoms corresponding to the expanded BEFAST framework, including sudden imbalance and visual disturbance, were identified by 47.5 and 43.2% of participants, respectively. Severe headache was recognized by 52.6% of respondents. Statistical comparison showed significantly higher recognition of traditional FAST symptoms compared with balance and vision symptoms (paired *t*-test, *p* < 0.001).

### 3.5 Attitudes toward emergency medical services

Attitudes toward emergency medical services revealed mixed response patterns among participants (Figure 3B). More than half of the participants (58%) reported they would call an ambulance in case of suspected stroke, while 21% preferred contacting a family member first. Thirteen percent stated they would go directly to the hospital without ambulance assistance, and 8% indicated they would wait until symptoms worsened. These findings suggest that although most respondents understood the importance of EMS activation, a substantial minority still relied on traditional or family-centered responses. This is concerning given that stroke is a time-critical condition where any delay significantly reduces the likelihood of effective thrombolysis. The reliance on family contact reflects cultural patterns commonly described in Asian populations, where family involvement in health decisions often supersedes individual action. Public health messaging may therefore need to target not only individuals but also families to reinforce the urgency of EMS activation. Full details are shown in Table 4.

**Table 4:** Help-seeking practices in prior stroke-like events (*n* = 600).

Response option	*N*	%
Call an ambulance (activate EMS)	348	58
Contact a family member first	126	21
Go directly to hospital without ambulance	78	13
Wait until symptoms worsened	48	8

### 3.6 Statistical analysis

**Figure 4:**
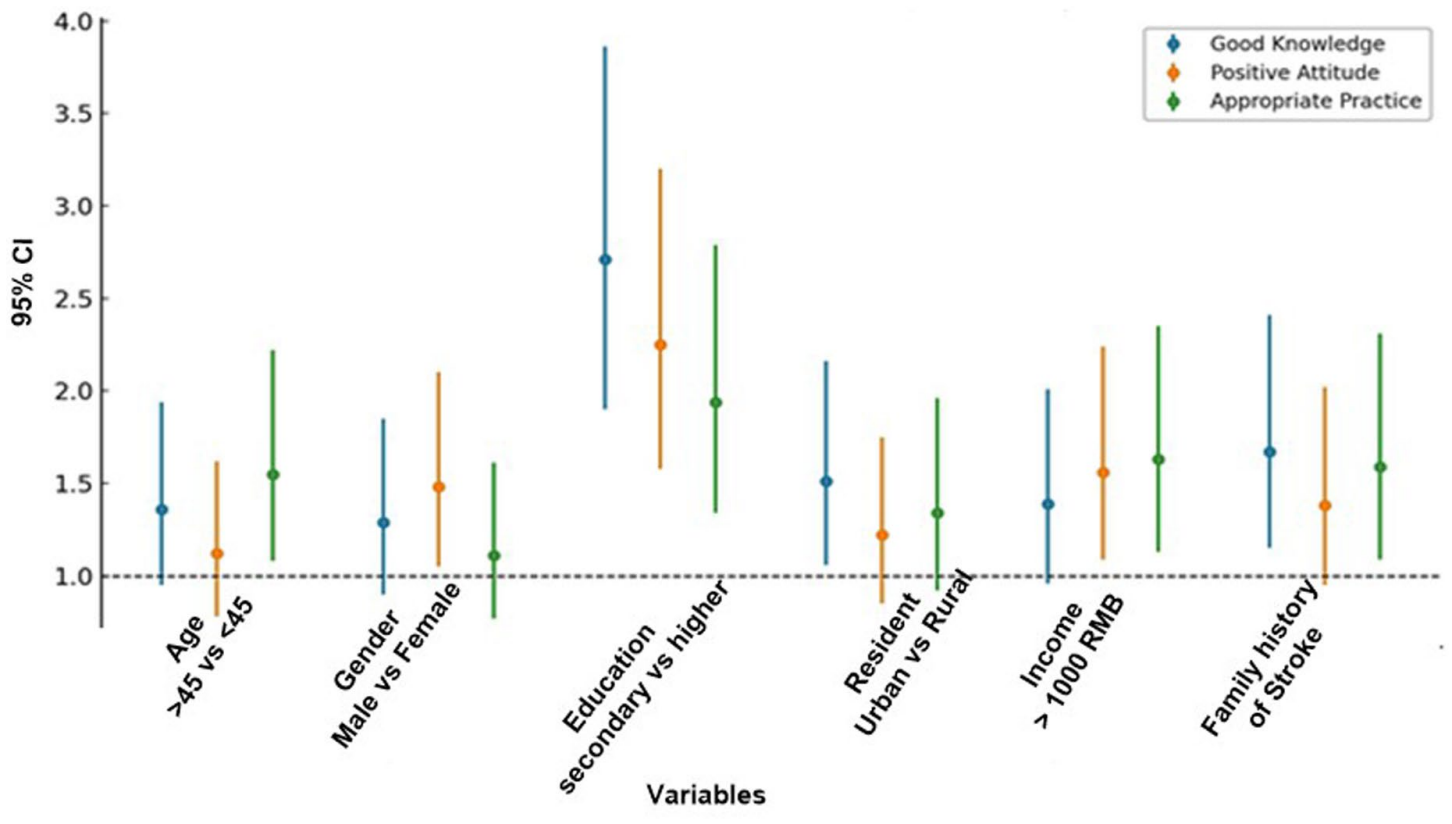
Logistic regression analysis of predictors of knowledge, attitudes, and practices (*n* = 600).

Comparative analysis of knowledge scores across demographic groups, Table 5 showed significant variation by age, education, and residence. One-way ANOVA revealed higher mean knowledge among participants aged 30–44 years (4.25 ± 1.70) compared with the youngest group (18–29 years, 3.95 ± 1.75; *p* = 0.042), while no differences emerged between middle-aged and older adults. Gender differences were not statistically significant (*p* = 0.18), though females consistently scored slightly higher across all domains. Education exerted the strongest influence (*p* < 0.001), with participants holding secondary or higher education achieving greater knowledge (4.55 ± 1.65) than those with lower education (3.40 ± 1.60). Residence was also important (*p* = 0.015), as urban residents reported better knowledge (4.30 ± 1.70) than rural counterparts (3.60 ± 1.85) (Figure 4).

**Table 5:** Summary statistics for KAP scores by demographic subgroup.

Group	*N*	Knowledge		Attitude		Practice		*p*-value (knowledge)
		Mean	SD	Mean	SD	Mean	SD	
All participants	600	4.10	1.80	1.75	0.85	1.55	0.95	–
Age 18–29	150	3.95	1.75	1.60	0.80	1.50	0.90	0.042
Age 30–44	180	4.25	1.70	1.85	0.84	1.65	0.95	
Age 45–59	150	4.05	1.85	1.70	0.88	1.45	0.95	
Age ≥60	120	4.10	1.95	1.80	0.90	1.50	1.0	
Male	320	4.05	1.85	1.70	0.86	1.50	0.96	0.18
Female	280	4.15	1.75	1.80	0.84	1.60	0.94	
Primary education	210	3.40	1.60	1.40	0.78	1.15	0.88	<0.001
Higher education	390	4.55	1.65	1.95	0.80	1.80	0.92	
Urban	420	4.30	1.70	1.85	0.83	1.65	0.93	0.015
Rural	180	3.60	1.85	1.45	0.80	1.25	0.98	

*p*-values from ANOVA or Kruskal–Wallis as appropriate; shown for knowledge to indicate subgroup differences.

### 3.7 Logistic regression analysis of predictors of knowledge, attitudes, and practices

Multivariable regression, Table 6 showed that education was the strongest independent predictor of favorable KAP outcomes. When KAP scores were analyzed, only 39% of respondents achieved a level defined as “good knowledge.” A slightly higher proportion, 52%, displayed positive attitudes toward emergency medical service (EMS) activation, while 45% demonstrated appropriate help-seeking practices. Higher education was associated with significantly better knowledge, positive attitudes, and appropriate practices. Older participants were more likely to adopt appropriate practices, while women demonstrated more positive attitudes than men. Urban residence predicted higher knowledge but not consistently better attitudes or practices.

**Table 6:** Logistic regression analysis of predictors of knowledge, attitudes, and practices (*n* = 600).

Variable	Good knowledge			Positive attitude			Appropriate practice		
	OR	95% CI	*P*	OR	95% CI	*P*	OR	95% CI	*P*
Age ≥45 vs. <45	1.36	0.95–1.94	0.09	1.12	0.78–1.62	0.52	1.55	1.08–2.22	0.02*
Female vs. Male	1.29	0.90–1.85	0.16	1.48	1.05–2.10	0.03*	1.11	0.77–1.61	0.56
Education ≥ secondary	2.71	1.90–3.86	<0.001*	2.25	1.58–3.20	<0.001*	1.94	1.34–2.79	<0.001*
Urban vs. Rural	1.51	1.06–2.16	0.02*	1.22	0.85–1.75	0.28	1.34	0.92–1.96	0.12
Income ≥5,000 RMB	1.39	0.96–2.01	0.08	1.56	1.09–2.24	0.01*	1.63	1.13–2.35	0.009*
Family history of stroke	1.67	1.15–2.41	0.007*	1.38	0.95–2.02	0.09	1.59	1.09–2.31	0.016*

*Statistically significant at *p* < 0.05; OR = odds ratio; 95% CI = 95% confidence interval.

Higher income correlated with positive attitudes and practices, underscoring socioeconomic influences on health behaviors. Family history of stroke was linked with better knowledge and greater likelihood of acting appropriately in suspected stroke events. The results suggest a gap between awareness and actual behavior, as the proportion with positive attitudes was larger than the proportion with good knowledge or practices. This mismatch indicates that although some participants valued rapid response, they lacked either the knowledge to recognize symptoms or the confidence to act promptly. These findings parallel community-based surveys, where knowledge levels are consistently below 50%, underscoring the need for greater public health education.

### 3.8 Information sources and delaying response on stroke

**Figure 5:**
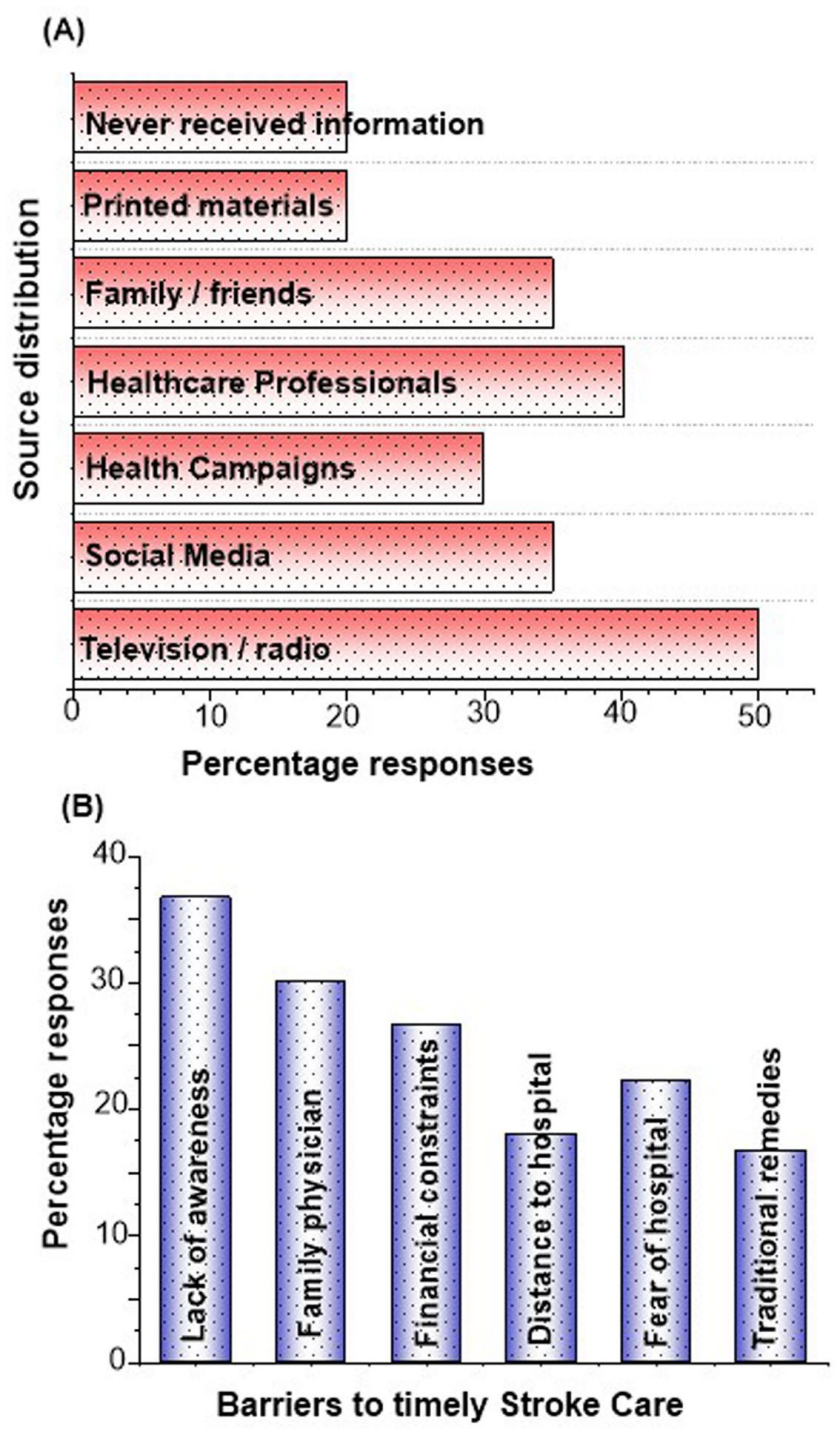
**(A)** Responses for information sources for stroke and **(B)** barriers to timely stroke care.

Television and radio were the most frequently reported sources of information on stroke (Supplementary Table S3), cited by half of the participants (50.0%) (Figure 5A). Healthcare professionals were the second most common source (40.2%), followed by social media or internet platforms and family or friends, both at 35.0%. Community-based health campaigns were reported by 30.0%. Printed materials, such as leaflets, were less common (20.0%), and the same proportion (20.0%) indicated they had never received any information about stroke. These findings suggest that mass media remains the primary channel of awareness, while direct educational interventions and printed resources are underutilized. The most commonly reported barrier to timely stroke care (Supplementary Table S4), was lack of awareness (36.7%) (Figure 5B), followed by limited access to a family physician or primary care provider (30.0%) and financial constraints (26.7%). Fear of hospitals (22.3%) and distance to healthcare facilities (18.0%) were also noted, while preference for traditional remedies was the least frequent barrier (16.7%).

## 4 Discussion

The demographic and clinical characteristics highlight that the study population is at moderate to high risk for cerebrovascular disease. The mean age of 51 years is consistent with the age at which stroke incidence begins to rise sharply in China. The relatively high prevalence of hypertension (32%) and diabetes (24%) in this sample underscores the need for preventive education in populations already burdened with vascular risk. Prior research in China has shown that individuals with comorbidities are more likely to encounter stroke, but paradoxically not always more aware of warning signs (10). The fact that 18% of participants had a previous stroke yet did not universally demonstrate good knowledge emphasizes that personal experience alone does not ensure awareness (9, 11). These findings suggest that risk factor–targeted health campaigns, rather than general public campaigns alone, could be more effective in reaching vulnerable populations.

Another result of interest was that a significant percentage of participants with a history of stroke in the past did not show the best understanding of stroke warning signs and emergency management process. This note indicates the possibility of a lapse in post-discharge education and secondary prevention counseling (12). Even though hospital-based treatment is mainly based on the acute management, long-term results also require that a patient should be capable of identifying recurring symptoms and seek immediate medical attention. The stroke education in a follow-up visit can be reinforced, caregiver-centered counseling can be introduced, and community-based survivor support programs can be developed to enhance the long-term awareness in the high-risk groups.

The analysis of socio-demographic subgroups revealed significant associations between KAP scores and factors such as education and gender, highlighting clear disparities across the population (7, 9). Participants with higher education demonstrated the most favorable outcomes, with 80% achieving high KAP scores, confirming a strong association between education and health literacy (*p* < 0.001). Those with secondary education performed moderately, with 55.6% in the high KAP category (*p* = 0.012), suggesting some benefit but still a clear gap compared with highly educated individuals. In contrast, respondents with primary education or less showed the poorest outcomes, with 58.3% falling into the low KAP category (*p* = 0.021), reinforcing that limited education remains a major barrier to stroke awareness and timely help-seeking (13). Gender differences were also notable. Although men showed a lower proportion of high KAP (56.3%) and the association was not statistically significant (*p* = 0.087), women reported markedly better outcomes, with 75% achieving high KAP (*p* = 0.039). This pattern reflects evidence that women often play a central role in health-related decision-making and may be more engaged with health information.

The fact that more than 60% of respondents recognized facial weakness but fewer than 30% recognized visual disturbances or severe headache illustrates a selective knowledge pattern that can be dangerous. Public campaigns often emphasize the FAST acronym (Face, Arm, Speech, Time), which is effective for recognizing common signs but less effective at communicating atypical symptoms. In this study, respondents’ recognition mirrored those campaigns, showing strong awareness of paralysis and speech loss but weaker recognition of other neurological deficits (14). This incomplete knowledge may contribute to delays in diagnosis when strokes present atypically, such as with visual symptoms or headache. In China, previous reports confirm that low awareness of non-motor symptoms contributes to delayed hospital arrival beyond the therapeutic window. Our findings support the recommendation that educational interventions broaden their focus to include the full range of symptoms, not only those emphasized by FAST campaigns.

Balance and visual disturbances recognition were significantly less than traditional FAST symptom recognition. This trend could also indicate a historical trend toward the popularization of the FAST mnemonic (Face–Arm–Speech–Time) in general awareness of stroke (15, 16). Conversely, the BEFAST method with the addition of Balance impairment and Eye symptoms to the symptom recognition expanded has not been introduced into educational projects as recently. New clinical findings indicate that BEFAST is a better approach to detecting the strokes of the posterior circulation that are sometimes overlooked under the FAST requirements only. Nevertheless, the comparatively less recognition that is witnessed in this research implies that the spread of BEFAST ideas in community education programs is still poor. Public health messaging can be extended to cover these other symptoms to enhance the early detection of stroke and prompt emergency switching (17–19).

Although 58% of participants indicated they would call an ambulance, 42% would not take the recommended immediate action, with 21% relying on family members and 13% opting for self-transport. Additionally, 8% reported they would wait until symptoms worsened. These findings reflect cultural and systemic barriers, including family-centered decision-making, perceived ambulance costs, doubts about availability, and limited understanding of stroke progression (20–22). Despite generally positive attitudes toward emergency response, a substantial proportion preferred non-emergency actions, such as consulting traditional healers or delaying care. Similar patterns have been observed in Chinese populations, where delayed EMS activation is linked to uncertainty about symptom severity and limited trust in emergency services (23–26). These results highlight those interventions must go beyond awareness-raising, addressing trust, access, and culturally influenced decision-making. Engaging family members, community leaders, and traditional healers in targeted campaigns may improve timely and appropriate EMS use.

Analysis of KAP scores using ANOVA and *t*-tests revealed that demographic factors, particularly education and residence, significantly influence knowledge of stroke warning signs and related practices. Participants aged 30–44 years demonstrated higher scores, likely reflecting greater exposure to health information and caregiving responsibilities, while differences between middle-aged and older adults were minimal. Gender differences were not statistically significant, although women showed slightly higher scores, possibly due to their role in family health management. Education was the strongest predictor, with higher-educated participants consistently achieving better knowledge, attitudes, and practices, supporting the role of education in enhancing health literacy and timely response to stroke. Urban residents outperformed rural participants across all KAP domains, highlighting disparities in healthcare access, information dissemination, and community awareness (9, 13). These findings emphasize the need for targeted interventions prioritizing individuals with lower education and rural residency to improve stroke recognition, promote timely help-seeking, and reduce prehospital delays (13).

The distribution of information sources, Figure 5A helps explain the observed variations in KAP toward stroke in Supplementary Table S2 (27). Mass media, reported by 50% of participants, appears effective in raising basic awareness but may not provide sufficient depth to translate knowledge into consistent practices, as reflected in the lower proportion of respondents with appropriate help-seeking behaviors (23, 28, 29). Healthcare professionals were cited by 40.2% of participants, and those exposed to direct medical guidance were more likely to demonstrate good knowledge and appropriate practices, supporting the role of trusted professional advice in improving stroke literacy. Social media and family or friends, each reported by 35%, likely contributed to shaping attitudes, but the risk of incomplete or inaccurate information may explain the mismatch between relatively positive attitudes (40%) and lower practice levels (30%) (23). The 20% who reported never receiving any information largely align with groups showing poor knowledge and inappropriate practices, particularly among those with lower education and rural residence (23, 27, 30). These findings suggest that while media campaigns can raise visibility, sustainable improvements in stroke preparedness require stronger engagement of healthcare professionals, community campaigns, and tailored outreach to populations currently left behind.

Barriers analyzed in Supplementary Table S4 provide deeper insight into structural and perceptual impediments to timely stroke care. The most frequently reported barriers (Figure 5B), lack of awareness of symptoms, financial cost, transport and distance, fear of hospital procedures, and reliance on home or traditional remedies, mirror those documented in the study from Huanggang, which showed that in rural regions, both knowledge deficits and logistics (distance, ambulance availability) significantly reduced EMS use (31). Moreover, in Taizhou, a survey of stroke patients found that prevention practices were undermined by cost and limited access to health education (14, 32, 33). These findings reinforce that improving knowledge alone is inadequate: policy must concurrently reduce financial, logistical, and cultural obstacles to empower people to act quickly.

Despite the fact that the current research has been carried out in the context of a Chinese hospital catchment region, the cases of a lack of knowledge about stroke symptom recognition have been also witnessed at the international level. In Europe, North America, and Australia, there is also evidence of the lower public awareness of the symptoms of stroke in the posterior circulation, including the problem of balance and visual impairment. These similarities indicate that the partial awareness of non-traditional stroke symptoms is not a regional problem but a worldwide issue of concern of the population. The inclusion of international evidence in stroke education programs can thus boost the efficiency of the public awareness programs.

## 5 Limitations

This study has several limitations. First, it was conducted at a single center with a relatively modest sample size, which may limit generalizability to broader populations. Second, although content validity and pilot testing were conducted, the questionnaire did not undergo full psychometric validation including confirmatory factor analysis, assessment of leading item effects, or advanced response-quality safeguards such as attention checks, instructed-response items, or negatively keyed control questions. Consequently, some knowledge items may have been susceptible to wording effects, and inattentive responding may not have been adequately detected. Despite these limitations, the study provides useful baseline evidence on stroke awareness and care-seeking behavior in a defined hospital catchment population.

## 6 Conclusion

Stroke remains a leading cause of death and long-term disability worldwide, yet timely recognition and response can dramatically improve outcomes. The findings of this study indicate that while a substantial proportion of participants (68%) could correctly identify at least two stroke warning signs, significant gaps persist in knowledge, attitudes, and practices related to stroke management. Only 55% expressed confidence in activating emergency services, and just 42% reported engaging in appropriate help-seeking behavior during a suspected stroke event, highlighting that awareness does not always translate into effective action. Higher educational attainment and prior exposure to stroke cases were significantly associated with better KAP scores (*p* < 0.05), suggesting that experience and education are key factors in preparedness. These results emphasize the need for structured community-based education and targeted interventions to improve both symptom recognition and prompt engagement with emergency care. Strengthening such initiatives can facilitate earlier treatment, reduce preventable complications, and ultimately lower morbidity and mortality associated with stroke.

## Data Availability

The original contributions presented in the study are included in the article/Supplementary material, further inquiries can be directed to the corresponding author.
