# Supplementary material for: Knowledge of stroke warning signs, attitudes to emergency activation, and help-seeking practices among adults in a hospital catchment: a single-center KAP study
Source: Front Public Health. 2026 Apr 24;14:1712520. doi: 10.3389/fpubh.2026.1712520 (PMC13153054; doi:10.3389/fpubh.2026.1712520)
Supplement: Supplementary file 1 [file Table_1.DOCX]

**Knowledge of Stroke Warning Signs, Attitudes to Emergency Activation, and Help-Seeking Practices among Adults in a Hospital Catchment: A Single-Centre KAP Study**

**Supplementary Data**

Table S1: A self-designed questionnaire

| **Section** | **Item** | **Question** | **Response Options** | **Scoring** |
| --- | --- | --- | --- | --- |
| **A. Demographic Information** | A1 | Age (years) | ___ (open response) | Not scored |
|  | A2 | Sex | ☐ Male ☐ Female ☐ Other | Not scored |
|  | A3 | Education level | ☐ No formal education ☐ Primary ☐ Secondary ☐ College/University ☐ Postgraduate | Not scored |
|  | A4 | Place of residence | ☐ Urban ☐ Rural | Not scored |
|  | A5 | Occupation | ☐ Unemployed ☐ Student ☐ Skilled worker ☐ Professional ☐ Retired ☐ Other (specify) ___ | Not scored |
| **B. Knowledge of Stroke** | B1 | Have you ever heard of stroke? | ☐ Yes ☐ No | Not scored |
|  | B2 | Which of the following do you recognize as warning signs of stroke? *(Multiple responses allowed)* | ☐ Sudden weakness/numbness of face, arm, or leg ☐ Sudden speech difficulty ☐ Sudden vision problems ☐ Sudden dizziness, loss of balance ☐ Sudden severe headache ☐ Do not know | Each correct option = 1 point (Max = 5) |
|  | B3 | What is the main risk factor you associate with stroke? | ☐ High blood pressure ☐ Smoking ☐ Diabetes ☐ High cholesterol ☐ Do not know | Correct = 1 point |
|  | B4 | Do you know the national emergency number to call for ambulance services? | ☐ Yes, specify: ___ ☐ No | Correct = 1 point |
| **C. Attitudes toward Stroke Response** | C1 | If you see someone suddenly unable to speak or move, what would you think it is? | ☐ Stroke ☐ Heart attack ☐ Do not know ☐ Other, specify ___ | Stroke = 1 point |
|  | C2 | How serious do you think stroke is as a medical condition? | ☐ Not serious ☐ Moderately serious ☐ Very serious ☐ Life-threatening | “Very serious” or “Life-threatening” = 1 point |
|  | C3 | If a family member develops sudden facial drooping and slurred speech, what would you advise first? | ☐ Call an ambulance immediately ☐ Take them to nearest hospital ☐ Wait and observe ☐ Use home remedies ☐ Do not know | Call ambulance / Hospital = 1 point |
| **D. Practices and Help-Seeking Behavior** | D1 | Have you ever witnessed someone with sudden stroke-like symptoms? | ☐ Yes ☐ No | Not scored |
|  | D2 | If yes, what was done first in that situation? | ☐ Called ambulance ☐ Took patient directly to hospital ☐ Consulted local doctor ☐ Waited at home ☐ Other, specify ___ | Ambulance / Hospital = 1 point |
|  | D3 | In case you personally develop sudden symptoms of stroke, what would be your first action? | ☐ Call ambulance ☐ Go to hospital ☐ Inform family/friends ☐ Use home remedy ☐ Do not know | Call ambulance / Hospital = 1 point |
|  | D4 | Have you ever participated in any stroke awareness campaign or health education program? | ☐ Yes ☐ No | Yes = 1 point |

**Table S2: Distribution of KAP levels by demographic characteristic**

| **Variable** | **Category (n)** | **Knowledge** | | | **Attitude** | | | **Practice** | | | **p-value*** |
| --- | --- | --- | --- | --- | --- | --- | --- | --- | --- | --- | --- |
|  |  | Level | n | (%) | Level | n | (%) | Level | n | (%) |  |
| **Total sample** | All (600) | Poor | 120 | 20 | Negative | 90 | 15 | Inappro-priate | 150 | 25 | — |
|  |  | Moderate | 330 | 55 | Neutral | 270 | 45 | Moderate | 270 | 45 |  |
|  |  | Good | 150 | 25 | Positive | 240 | 40 | Appropriate | 180 | 30 |  |
| **Age** | 18–29 (150) | Poor | 30 | 20 | Negative | 15 | 10 | Inappropriate | 30 | 20 | 0.001 |
|  |  | Moderate | 85 | 56 | Neutral | 75 | 50 | Moderate | 75 | 50 |  |
|  |  | Good |  |  | Positive |  |  | Appropriate |  |  |  |
|  | 30–44 (180) | Poor | 30 | 16 | Negative | 20 | 11 | Inappropriate | 36 | 20 |  |
|  |  | Moderate | 98 | 54 | Neutral | 76 | 42 | Moderate | 84 | 46 |  |
|  |  | Good | 52 | 29 | Positive | 84 | 46 | Appropriate | 60 | 33 |  |
|  | 45–59 (150) | Poor | 36 | 24 | Negative | 30 | 20 | Inappropriate | 45 | 30 |  |
|  |  | Moderate | 79 | 52 | Neutral | 66 | 44 | Moderate | 66 | 44 |  |
|  |  | Good | 35 | 23 | Positive | 54 | 36 | Appropriate | 39 | 26 |  |
|  | ≥60 (120) | Poor | 24 | 20 | Negative | 25 | 20 | Inappropriate | 39 | 32 |  |
|  |  | Moderate: 68 (56.7%) | 68 | 56 | Neutral: 53 (44.2%) | 53 | 44 | Moderate: 45 (37.5%) | 45 | 37 |  |
|  |  | Good | 28 | 23 | Positive | 42 | 35 | Appropriate | 36 | 30 |  |
| **Sex** | Male (320) | Poor | 68 | 21 | Negative | 48 | 15 | Inappropriate | 80 | 15 | 0.040 |
|  |  | Moderate | 176 | 55 | Neutral | 144 | 45 | Moderate | 144 | 45 |  |
|  |  | Good | 76 | 23.8 | Positive | 128 | 44 | Appropriate | 96 | 30 |  |
|  | Female (280) | Poor | 52 | 18 | Negative | 42 | 15 | Inappropriate | 70 | 25 |  |
|  |  | Moderate | 154 | 55 | Neutral | 26 | 45 | Moderate | 126 | 45 |  |
|  |  | Good | 74 | 26 | Positive | 112 | 40 | Appropriate | 84 | 30 |  |
| **Education level** | No formal (60) | Poor | 28 | 46 | Negative | 20 | 33 | Inappropriate | 30 | 50 | <0.001 |
|  |  | Moderate | 26 | 43 | Neutral | 30 | 50 | Moderate | 20 | 33 |  |
|  |  | Good | 6 | 10 | Positive | 10 | 16 | Appropriat | 10 | 16 |  |
|  | Primary (120) | Poor | 36 | 30 | Negative | 24 | 20 | Inappropriate | 36 | 30 |  |
|  |  | Moderate | 64 | 53 | Neutral | 60 | 50 | Moderate | 60 | 50 |  |
|  |  | Good | 20 | 16 | Positive | 36 | 30 | Appropriate | 24 | 20 |  |
|  | Secondary (210) | Poor | 36 | 17 | Negative | 30 | 14 | Inappropriate | 45 | 21 |  |
|  |  | Moderate | 117 | 55 | Neutral | 93 | 44 | Moderate | 93 | 44 |  |
|  |  | Good | 57 | 27 | Positive | 87 | 41 | Appropriate | 72 | 34 |  |
|  | College/University (160) | Poor | 16 | 10 | Negative | 10 | 6.2 | Inappropriate | 21 | 13 |  |
|  |  | Moderate | 86 | 53 | Neutral | 60 | 27 | Moderate | 64 | 40 |  |
|  |  | Good | 58 | 36 | Positive | 90 | 56 | Appropriate | 75 | 46 |  |
|  | Postgraduate (50) | Poor | 4 | 8 | Negative | 6 | 12 | Inappropriate | 6 | 12 |  |
|  |  | Moderate | 37 | 74 | Neutral | 12 | 24 | Moderate | 33 | 66 |  |
|  |  | Good | 9 | 18 | Positive | 32 | 64 | Appropriate | 11 | 22 |  |
| **Residence** | Urban (420) | Poor | 72 | 17 | Negative | 48 | 11 | Inappropriate | 90 | 21 | 0.020 |
|  |  | Moderate | 231 | 55 | Neutral | 189 | 45 | Moderate | 189 | 45 |  |
|  |  | Good | 117 | 27 | Positive | 183 | 43 | Appropriate | 141 | 33 |  |
|  | Rural (180) | Poor | 48 | 26 | Negative | 42 | 23 | Inappropriate | 60 | 33 |  |
|  |  | Moderate | 99 | 55 | Neutral | 81 | 45 | Moderate | 81 | 45 |  |
|  |  | Good | 33 | 18 | Positive | 57 | 31 | Appropriate | 39 | 21 |  |

**Table S3 Sources of stroke information reported by participants (multiple responses allowed; n = 600)**

| **Information source** | **Reported Frequency**  **(n)** | **%** | **95% CI** |
| --- | --- | --- | --- |
| Television / radio | 300 | 50.0 | 46.2–53.8 |
| Social media / internet | 210 | 35.0 | 31.5–38.7 |
| Health campaigns / community talks | 180 | 30.0 | 26.6–33.6 |
| Healthcare professionals | 240 | 40.2 | 36.4–43.7 |
| Family / friends | 210 | 35.0 | 31.5–38.7 |
| Printed materials (leaflets) | 120 | 20.0 | 17.0–23.4 |
| Never received any information | 120 | 20.0 | 17.0–23.4 |

Notes: counts reflect number of participants endorsing each source; items non-exclusive

**Table S4. Reported barriers to timely stroke response (n = 600).**

| **Barrier** | **n** | **(%)** |
| --- | --- | --- |
| Lack of awareness | 220 | 36.7 |
| Family physician/Primary Care | 180 | 30.0 |
| Financial constraints | 160 | 26.7 |
| Distance to hospital | 120 | 18.0 |
| Fear of hospital | 140 | 22.3 |
| Preference for traditional remedies | 100 | 16.7 |
